# Cross-Sectional Analysis of Family Factors Associated with Lifestyle Habits in a Sample of Italian Primary School Children: The I-MOVE Project

**DOI:** 10.3390/ijerph20054240

**Published:** 2023-02-27

**Authors:** Francesco Sanmarchi, Alice Masini, Carolina Poli, Anna Kawalec, Francesco Esposito, Susan Scrimaglia, Lawrence M. Scheier, Laura Dallolio, Rossella Sacchetti

**Affiliations:** 1 Department of Biomedical and Neuromotor Science, Alma Mater Studiorum, University of Bologna, 40126 Bologna, Italy; 2 Department of Medical and Surgical Science, Alma Mater Studiorum, University of Bologna, 40126 Bologna, Italy; 3 Department and Clinic of Paediatric Nephrology, Wroclaw Medical University, 50-556 Wroclaw, Poland; 4 LARS Research Institute, Inc., Sun City, AZ 85351, USA; 5 Prevention Strategies, Greensboro, NC 27410, USA; 6 Department of Education Studies “Giovanni Maria Bertin”, Alma Mater Studiorum, University of Bologna, 40126 Bologna, Italy

**Keywords:** diet quality, healthy lifestyles, children, leisure screen time, sedentary behavior

## Abstract

The acquisition of healthy dietary and exercise habits during childhood is essential for maintaining these behaviors during adulthood. In early childhood, parents have a profound influence on a child’s lifestyle pursuits, serving as both role models and decision-makers. The present study examines family factors as potential contributors to healthy lifestyle habits and their child’s overall diet quality among a sample of primary school children. A secondary aim is to evaluate several aspects of diet quality using the Mediterranean adaptation of the Diet Quality Index-International (DQI-I). This cross-sectional study involved 106 children enrolled in a primary school located in Imola, Italy. Data were collected from October to December 2019 using an interactive tool used to assess parent characteristics, children’s lifestyle, food frequency (ZOOM-8 questionnaire), and actigraph accelerometers to capture children’s physical activity and sedentary behavior. Adherence to the Mediterranean Diet (expressed by KIDMED Index) was positively associated with fathers’ educational level, parental sport participation, and the parent’s overall nutritional knowledge. Higher mothers’ educational level was inversely associated with children’s leisure screen time. Parents’ nutritional knowledge was positively related to children’s average daily minutes of organized sport activities. The better score for DQI-I was for consumption adequacy, followed by variety and moderation. The lowest score was for overall balance. The present study reinforces the importance of family factors in young children’s lifestyle choices, particularly their dietary, leisure time, and exercise habits.

## 1. Introduction

An imbalance between calorie intake and expenditure, often due to unbalanced diets high in saturated fats, trans fatty acids, sugar and salt, as well as excessive sedentary behavior contribute to numerous adverse health effects (e.g., increased adiposity; poorer cardiometabolic health, fitness, behavioral conduct/prosocial behavior, and reduced sleep duration) [1]. Healthy nutrition is defined as the intake of an adequate and well-balanced diet to support the body’s energy needs, and a healthy lifestyle is characterized by physical activity (PA) and other behaviors that promote overall health and well-being [2]. In recent decades, there has been a transition from traditional, healthier dietary patterns such as the intake of fruits, vegetables, whole grains, and lean protein sources, as well as limited intake of added sugars, saturated and trans fats, and sodium) [2] to less healthy ones, particularly among young children, which has greatly affected diet quality [3,4]. Despite this trend, the Mediterranean Diet (MD), in particular, has received significant attention due to its demonstrated protective effects against metabolic risk, cardiovascular disease, and various types of cancer [5,6,7].

The MD is characterized by a high intake of fruits and plant-based foods (i.e., legumes, nuts, and seeds), unprocessed cereals, extra-virgin olive oil, dairy products (milk, yogurt, and cheese), low to moderate intake of fish and poultry meat, and reduced consumption of red meat [8]. These dietary ingredients have been shown to be beneficial for countering the risk of developing various diseases (e.g., cardiovascular, endocrine, and psychiatric conditions), improving physical and mental health [9,10], and increasing lifespan [11,12]. Recent studies have specifically confirmed numerous benefits of the MD for children and adolescents [13].

In light of this evidence, a growing number of literature reviews [14,15] and published guidelines [14,16] have suggested the importance of implementing health promotion interventions targeting young children in primary school settings [17]. Physical activity and nutrition are crucial components of healthy lifestyle interventions, as they provide essential protective measures that can positively guide children’s health and development [18]. In contrast, the absence of PA and poor dietary quality are significant determinants of chronic diseases and further compound associated risk factors, including childhood and adolescent obesity [19,20]. The World Health Organization (WHO) has recommended that children and adolescents should perform at least 60 min of moderate-to-vigorous PA (MVPA) per day in order to avoid the risk of metabolic and cardiovascular diseases. Additionally, according to recent WHO guidelines [21], children and adolescents should limit the time they spend engaging in sedentary activities in order to reap physical and mental health benefits. Sedentary behavior (SB) is defined as any waking behavior that is performed while sitting, reclining, or lying down with low energy expenditure [22]. Included in this definition is the recreational use of computers and television viewing, activities that require little sustained physical effort. This type of behavior is prevalent and pervasive in developed countries [23] and has been linked to negative health outcomes such as an increased risk of type 2 diabetes, cardiovascular disease, all-cause mortality in adults, and a reduction of sleep duration, reduced prosocial behaviors and increased depressive symptoms in children and adolescents as well as increased adiposity and poorer cardiometabolic health [24,25]. According to the last available Childhood Obesity Surveillance Initiative (COSI) round, after Cyprus and Greece, Italy was the country with the highest both obesity and overweight prevalence in children. In particular, in the Emilia-Romagna region, the prevalence of overweight children was 20.4%, while the prevalence of obesity and severe obesity was, respectively, 6.9% and 2.4%. Although there has been a slight reduction over the years of all morbidity indicators, overweight and obesity remains an important public health issue. Furthermore, only 6 in 10 children spend less than two hours a day watching TV, video games, tablets and mobile phones and 16% did not have any physical activity the day before the survey [26].

A child’s rearing environment has an important influence on their choice of a healthy lifestyle [27,28]. Two prominent sources of influence that make up this rearing environment include their family and school [29]. The family (i.e., parents and caregivers) plays a prominent role because they can transmit beliefs and attitudes that support a positive outlook on healthy nutrition and dietary practices as well as convey important cultural and emotional ties to food. This includes extolling the importance and favorable outcomes of health living. Consistent reinforcement of healthy living can have a tremendous influence on a child’s healthy lifestyle pursuits including selection of foods to eat, consumption patterns, and whether a child will engage in PA [30,31,32]. When parents are active as part of their own lifestyle pursuits and routinely eat healthy foods, they serve as role models to their children, who engage in similar health-engendering behaviors. Children can learn vicariously from their parents by watching them shop at the supermarket and also viewing food preparation in the home. They can also assist their parents in food preparation and thus learn directly by monitoring what their parents provide for breakfast, lunch, and dinner as well as the different kinds of snacks they are offered. Parents can also encourage children to actively play sports and motivate them through family activities that center on athletic endeavors. Added to the family’s influence, schools are also a place where children can learn about healthy lifestyles including what foods are available in the cafeteria for lunch and the school’s reinforcement of PA as part of a child’s physical education [33]. Children and adolescents spend a significant portion of their daily time in school and are influenced by their physical and social environments, such as school health policies, nutrition education and support, and physical education [34]. In this respect, schools are an ideal setting for reaching as many children as possible, regardless of gender, socio-economic status, or background, and represent a favorable environment for reducing health inequalities.

While most school-based interventions aimed at promoting healthy lifestyles have focused solely on the school setting, some reviews have found that multicomponent interventions involving the family, in addition to the school, are likely to be the most effective [35]. Previous studies have demonstrated the significant influence of parents on the PA patterns and food intake of young people, as well as their role in promoting behavior change [36]. Of note, few studies have investigated these associations in an Italian population of primary school children, presenting mixed results [37]. With this in mind, the current study examines family factors as potential correlates of healthy lifestyle habits (adherence to the MD, leisure screen time, moderate to vigorous PA, and engagement in organized sports) among primary school children living in Bologna, Italy. A secondary aim is to evaluate several aspects of diet quality using the Mediterranean adaptation of the Diet Quality Index-International (DQI-I) [38] in a sample of primary school children. The study offers a novel use of assessment strategies to map the influence of family factors on children’s healthy lifestyles and dietary habits.

## 2. Materials and Methods

### 2.1. Study Design and Participants

This cross-sectional study was conducted with a sample of children enrolled in the “I-MOVE” project [39], with all children from a primary school in Imola (70,075 inhabitants, Bologna, Italy), located in the Emilia Romagna region of northeastern Italy. The Bioethics Committee of the University of Bologna approved the “I-MOVE” project on 18 March 2019 (approval number: 0054382), and the study was conducted in accordance with the Declaration of Helsinki. The research team obtained written informed consent from the parents of participating children. Invitation letters were sent to the principals of primary schools located in Imola. One school expressed interest in participating in the I-MOVE project and 10 teachers representing 5 classes agreed to participate. Children from these classes were then recruited if their teachers provided access, parents consented, and the child assented to participate.

Children were recruited to participate from the first to fifth grades (corresponding to elementary school) and inclusion criteria included not having any health issues or physical disabilities that might interfere with or impact their PA performance. Children in primary school in Italy attended 2 h per week of physical education lessons. Children normally had lunch in the school’s canteen. Therefore, examining parental influence on dietary practices refers only to dinners and weekends’ meals. The study was designed based on the Strengthening the Reporting of Observational Studies in Epidemiology (STROBE) reporting guidelines [40].

### 2.2. Instruments

Data from parents and their children were collected from October to December 2019 using the ZOOM-8 questionnaire [41] and actigraph accelerometers worn by each child. The ZOOM-8 questionnaire is an interactive tool used to assess parental characteristics and parents’ reports of their child’s lifestyle (over the past year), including nutrient and food intake, PA levels, and sedentary behaviors [41]. The questionnaire was completed by a parent/caregiver, and was evaluated for accuracy and completeness by one of the investigators prior to data analysis. The ZOOM-8 questionnaire contains two parts:

- Part 1 includes items that assess the child’s lifestyle (e.g., leisure screen time, sleeping hours, participation in organized and structured sport activities) and characteristics of the parents (e.g., age, educational level, weight and height, PA, and nutritional knowledge).
- Part 2 consists of a semiquantitative food frequency questionnaire (FFQ), following the methodology described and validated by Willett [42]. The instrument consists of 53 commonly used food items categorized into 11 food groups [43]. Frequency response categories for all food servings ranged from daily, weekly, monthly, annually, to never. A reference food was considered for different items, and photographs of different foods were provided to help respondents gauge portion-sizes.

Energy density, macro- and micro-nutrient intakes were calculated using the MetaDieta software Professional 4.0.1 (Me.Te.Da., San Benedetto del Tronto, Italy, 2019), which includes an Italian database of food composition.

Each child’ body max index (BMI) was calculated based on age and sex using International Obesity Task Force (IOTF) cut-off values [44,45], with children’s height and weight measured and recorded directly by the researchers. A dichotomous variable was created to indicate whether at least one of the parents engaged in PA at least once a week (yes/no).

The child’s PA levels and SB were monitored over a seven-day period using actigraph accelerometer models GT3X (ActiGraph LCC: Pensacola, FL, USA). The data were analyzed using ActiLife 6.13.3 software (ActiGraph LCC: Pensacola, FL, USA) with an epoch length of 10 s to allow for a detailed estimate of PA intensity [46]. Children wore the accelerometers around their waist with an elastic belt over a seven-day period (five weekdays and two weekend days), only removing them for bathing, swimming, and showering [47]. We analyzed the accelerometer’s data only when children complied with specific inclusion criteria: having worn the accelerometer on at least 3 weekdays and 1 weekend day, and for at least 10 h every day. Minutes spent in PA (light, moderate, and vigorous) per day were calculated using the Evenson cut-points [48].

### 2.3. Adherence to the Mediterranean Diet

We calculated adherence to the MD using the modified Mediterranean Diet Quality Index for Children and Adolescents (KIDMED) score [41,49]. We classified the children into three categories based on their KIDMED index: (1) high (KIDMED score 8–12), (2) medium (KIDMED score 4–7), and (3) poor adherence (KIDMED score 0–3). The KIDMED index provides an ideal means to assess MD adherence because it is easy to interpret and provides a comprehensive overview of an individual’s dietary intake.

### 2.4. Diet Quality Index-International (DQI-I)

The Diet Quality Index-International [50], represents one of the most thorough and validated instruments to assess diet quality. We used the adapted version for the Mediterranean population [38], which is consistent with other studies based in Italy [51]. The DQI-I explores four factors considered necessary for a healthy diet: variety, adequacy, moderation, and overall balance. Current dietary guidelines encourage adequate consumption of different food groups (variety) and key nutrients for health (adequacy, further suggesting moderation in the intake of certain food elements such as saturated fat, cholesterol, sodium and sugar, and a healthy proportion of macronutrients (overall balance) [2,52].

The four aspects of a child’s diet were evaluated as follow:

*Variety* (0–20 score)—Variety was evaluated both as overall variety among the five food groups (meat/poultry/fish/egg, dairy/beans, grains, fruit, and vegetables) and variety within the protein source groups (meat, poultry, fish, eggs, dairy, beans).

*Adequacy* (0–40 score)—This category evaluates the intake of dietary elements that must be consumed sufficiently to ensure a healthy diet. Adequacy was assessed considering daily intakes of fruits, vegetables, grains and fiber, protein, iron, calcium and vitamin C. Cut-off values derive from the frequencies of consumption and the daily intake recommended for Italian school children [52,53].

*Moderation* (0–30 score)—Moderation evaluates the intake of food and nutrients related to chronic diseases and that require a low level of consumption. Modification of the original DQI-I was used, according to Tur et al. [38], which suggests an increase optimally to <30% of total energy/d from fat (instead of 20%), due to presence of olive oil as main source of monounsaturated fatty acids (MUFA) in Mediterranean countries. Saturated fat intake was also assessed as the percentage of energy from saturated fat. Cholesterol and sodium intake levels were also calculated. The ‘empty-calorie food’ component assesses how much a person’s energy supply depends on low-nutrient density foods, which provide energy but insufficient nutrients (e.g., sugar, industrial pastries, sweets, and sugary drinks) [38].

*Overall balance* (0–10)—This category examines the overall balance of diet in terms of proportions of energy sources and fatty acid composition. The proposed cut-off points and corresponding scores reported by Tur et al. [38] were considered as more suitable for individuals residing in a Mediterranean country.

The score for each category is the sum of the scores for each component in that category. The total DQI-I score (0–100 scale) is the sum of the scores across the four categories. A score below 60 indicates a poor-quality diet [50].

### 2.5. Parental Nutritional Knowledge

Parental nutritional knowledge (NK) was calculated using seven questions extracted from the ZOOM-8 questionnaire [41]. The questions evaluated whether parents were aware of dietary recommendations for their children regarding breakfast, snack, beverage and vegetable intake (e.g., knowledge of the dietary recommended intake for vegetables, the importance of breakfast, the adequate breakfast and mid-morning snack and the appropriate beverages for school children). Each question was assigned “1” if the answer was correct and “0” if the answer was not correct. The correct answer was the one adhering to dietary recommendations. The final score comprised a unit-weighted index of knowledge ranging from 0 to 7.

### 2.6. Data Analysis

Estimation of the appropriate sample size was previously calculated for the primary analysis [39]. A post-hoc power analysis was conducted to determine if the current sample size was adequate to reliably estimate the multiple regression model presented. Continuous variables are described using mean and standard deviation (±SD) while categorical variables are described through absolute and relative frequencies. Normal distribution of dependent variables was assessed graphically using density graphs and tested with the Shapiro-Wilk test.

The outcome variables investigated in this study included adherence to the MD (KIDMED Index), average daily minutes of organized PA, average daily leisure screen time, and average daily minutes of moderate-vigorous PA (MVPA). The leisure screen time variable was dichotomized using 100 average daily minutes as the cut-off to address its bimodal distribution.

The associations between predictor variables and the designated outcomes were analyzed using Student’s t-test for continuous means or analysis of variance (ANOVA) when three or more levels were statistically compared. Multiple linear regression models with backward stepwise selection were employed to identify efficient predictors associated with the outcome measures. Results from linear regression were reported as unstandardized regression coefficients (*Beta*) with relative 95% confidence intervals (95%CIs).

The regression models were covariate-adjusted for child’s gender and age and the time of the survey and also included mother’s and father’s education level (high school or lower vs. university degree or higher), parent’s nutrition knowledge (0 to 7 scale), and parents’ PA levels (involved vs. uninvolved). The statistical significance level was set as *p* < 0.05. All analyses were carried out using R version 4.2.2 (R Project for Statistical Computing) [54].

## 3. Results

### 3.1. Population Characteristics

A total of 106 questionnaires were available for inclusion in the analyses. The sample included *n* = 53 girls (50%) and *n* = 53 boys (50%), between the ages of 6 to 10 years (mean 7.92 ± 1.40). Based on Cole cut-off values and also sex and age, more than half of the sample was categorized as normal-weight (*n* = 66; 63%) and the remaining portion was categorized as overweight/obesity (*n* = 39; 37%). The average KIDMED score was 4.44 ± 2.28. Specifically, *n* = 35 (33%) children reported low adherence to the MD, *n* = 59 (56%) intermediate adherence, and *n* = 12 (11%) reported optimal adherence. Children were engaged in organized sport activities on average for 22.46 ± 16.91 min daily, while the accelerometers reported 48.25 ± 17.74 min of daily average MVPA. The average daily leisure screen time was 102.31 min (SD = 33.84). Detailed sample characteristics are summarized in Table 1. Males spent more time involved in organized sports (See Supplementary Materials Table S2) and had higher mean levels of vigorous physical activity. The investigated variables were normally distributed except for average daily organized sport (minutes), which was positively skewed.

### 3.2. Regression Models

Table 2 contains the results of the multivariate linear regression models. Father’s educational level was positively associated with the KIDMED index (reference class = “High school or lower” β = 1.0; 95%CI 0.10, 1.9). Mother’s education, on the other hand, was negatively associated with leisure screen time (OR = 0.29; 95%CI 0.10, 0.81). Parental NK was positively associated with the KIDMED index (β = 0.45; 95%CI 0.10, 0.81), and likewise with involvement in sport activities (β = 2.8; 95%CI 0.15, 5.4). Parent’s sport engagement was positively related to the KIDMED score (β = 1.0; 95%CI 0.17, 1.9).

Older children spent more time engaged in leisure screen time (OR = 1.26; 95%CI 1.02, 1.73), were more time engaged in organized sports (β = 3.5; 95%CI 1.3, 5.7), and spent less time committed to vigorous physical activity (β = −25; 95%CI −42, −8.1). Male children spent more time engaged in organized sports (β = 8.1; 95%CI 2.0, 14) and more time in vigorous physical activity (β = 82; 95%CI 36, 129).

### 3.3. Diet Quality Index-International (DQI-I) Evaluations

Descriptively, the mean total DQI-I score for the complete sample was 53.89 ± 8.69 (on a scale of 0 to 100). Slightly less than one-quarter (24%, *n* = 27) of the children had a total DQI-I score higher than 60 (indicating an intermediate/good diet quality [50]). In terms of subdomains, the better score was for adequacy, followed by variety and moderation. The lowest score was for overall balance (Table 3). In terms of variety, only 4.5% of the children consumed at least one serving from each food type, 26% missed only one food group, and 39% missed two food groups. Additionally, only 1.8% of the sample consumed three or more different sources of protein per day. According to the adequacy category, the majority of the sample reported >50% of the recommended intake for grains, fiber, protein, iron, calcium, and vitamin C. Most children failed to meet the recommended levels of vegetables and fruit intake (Table 3). In the moderation category, only 5.4% and 0% of adolescents achieved the fat (≤30% of total energy intake) and saturated fat goals (≤7% of total energy intake), respectively. Cholesterol intake was ≤300 mg/day in 79% of the sample. A relatively low overall balance score was also found for macronutrients ratio, and among fatty acids ratio.

## 4. Discussion

In the current study, we used cross-sectional data obtained from the first wave of the I-MOVE study [39] in order to examine parental influences on children’s dietary and physical exercise practices. Importantly, we used several novel assessment strategies that provided much richer insight into children’s eating and exercise tendencies and linked this information to parental influences. Included in the assessment was the KIDMED [49] instrument to measure the child’s compliance with the MD, actigraph accelerometers to accurately measure physical activity, the ZOOM-8 [41] to assess nutrient and food intake, lifestyle factors and parents’ characteristics (i.e., nutritional knowledge and their own activity levels). Added to this, we were able to characterize the sample based on DQI-I scores [38] to assess the children’s diet quality and quantify their sedentary behavior. Taken as a whole, this wide array of assessments provides much richer insight into a child’s nutritional practices, their routine physical activity and compliance with the MD.

In terms of diet, DQI-I scores indicated that less than a quarter of the sample would be categorized as having an intermediate/good diet quality. Moreover, less than a quarter could be characterized as having diets that contained adequate balance, and a very small percentage had sufficient exposure to different food groups. The mean DQI-I score was slightly above 50%, in line with previous studies on children and adolescents from the Mediterranean region [51,55]. According to DQI-I scores obtained, the children in this sample had diets that lacked moderation in fat intake and were highly unbalanced towards saturated fats. Also, regarding the adequacy of their diets, most of the children did not meet the recommended daily intakes of fruit and vegetables. These findings are consistent with those reported in other Mediterranean populations [56]. These results can be explained by recent change in nutrition patterns, which has witnessed an increase in high-caloric, high fat foods and the worldwide problem of overnutrition [57]. With regard to PA levels, less than half (39%) of our sample met the WHO’s recommended criteria of at least 60 min of MVPA per day [58]. However, the majority of our sample (89%) reported participating in sports activities outside of school hours. In addition, when we examined self-reported sedentary behavior and, more specifically, leisure screen time, 42% of our sample exceeded 100 daily minutes.

To our knowledge only one other Italian study, conducted in Sicily with older youth [51], used the DQI-I index to characterize multiple facets of diet quality in Italian youth. By comparing DQI-I scores of our sample with those obtained from Ferranti et al. [51] we found that our sample had lower scores for the variety category including protein sources and fatty acid ratio scores. One explanation for these differences is the large amounts of fish consumed by individuals residing in southern Italy. Fish is an important source of proteins and polyunsaturated fatty acid in Mediterranean countries.

In our study, we found that parents’ education played a role in children’s compliance with the MD and also their leisure time (sedentary behavior). In particular, more educated fathers had children with greater adherence l to the MD and more educated mothers had more active children who engaged in less sedentary behavior.

Other studies, also conducted in Italy, have also found associations between parents’ education and improved adherence to the MD [59]. In particular, Grosso and colleagues [59] found that high socioeconomic status (combining education and occupation) was positively associated with MD adherence in a relatively large sample of adolescents recruited from schools located in the Sicily region (southern Italy). Likewise, additional studies conducted in other European countries [57,60,61,62] reinforce these findings. The connections between parental education and childhood dietary practices are not necessarily clear, but may involve social and cultural factors operating alone or in tandem. Parents with a higher level of education may be more concerned about their children’s weight and more knowledgeable about the benefits of exercise and nutritional eating habits, leading them to offer their children more chances to adopt a healthier way of life [63]. In our case, only fathers’ education was associated with MD adherence (mother’s education was significant in the univariate analyses, but dropped out in the multivariate analyses). Assortative mating may be one reason for these findings, given that educated fathers may select highly educated partners. Given the high magnitude of association between education levels, both scores cannot be efficiently estimated in a single regression model. Notwithstanding, in Italy, which from a culinary standpoint is a heavy matriarchal society, mothers play a prominent role in the lifestyle choices made by children. They are actively involved in food selection and preparation, and they traditionally spend more time with their children [64].

There are other more subtle relations that may lay at the heart of the education–diet and activity relationship. Education and socioeconomic status (SES) are also confabulated to some degree, as education provides a means for higher income. With more money available, parents can afford to enroll their children in afterschool programs including organized sports [65], spend more time with their children [66,67], and provide their children with healthier food choices [68].

A substantial literature has investigated the role of parental modeling on children’s lifestyles [69], with a particular focus on dietary habits and active behaviors [70]. The existing evidence is not definitive, as some previous studies have reported a positive association between the PA of parents and children [71], while others have not found any significant effect of parental PA on children’s physical fitness and engagement in organized sports [72,73]. Our findings align with those of Ruedl and colleagues [72], and support the hypothesis that parental influence on children’s PA may be mediated by the child’s age [74].

Both age and gender were important factors in dietary practices (adherence to MD) and physical activity. Older children and boys spent more time in organized sports, older children spent more time engaged in leisure activity, and accelerometer data showed that older children spent less time engaging in vigorous PA. These results suggest that developmental changes may be related to a child’s lifestyle [75] and are consistent with previous research indicating that older children tend to be more engaged in organized sports [76] and that boys tend to engage in higher levels of PA than girls [77]. Numerous factors may bear on whether a boy or girl plays organized sports or engages in PA. Individual-level factors can include a child’s body weight, overall fitness, boy’s preferences for higher intensity activities, family factors (e.g., parent’s support, gender roles, living conditions), community (e.g., participation in community sport), school (e.g., opportunities to be physically active in school) and environmental considerations (e.g., climate or geography) [78].

Parents with higher nutritional knowledge had children who adhered better to the MD and also spent more time actively involved in organized sports. Previous evidence suggests that parents with high nutritional knowledge are more likely to meet the preventive and health care needs of their children [79]. Furthermore, our findings align with Romanos-Nanclares and colleagues [80] who reported that children with parents having more favorable healthier-eating attitudes were less likely to present micronutrient inadequacy and higher adherence to the MD. Notably, the impact of nutritional knowledge scores on children’s PA was limited to minutes spent in organized sports, while it did not affect their MVPA, the latter assessed using accelerometers. Conceivably, schools might play a role in improving vigorous physical activity by delivering interventions focused on improving students’ physical fitness. The use of school-based interventions also goes a long way toward reducing social inequalities given that schools must offer programs irrespective of race, social class, and other potential factors (economic and cultural) that may serve as barriers to PA and dietary practices [81].

The early formative years of a child’s life are incredibly important toward shaping their future health and well-being. It is during this time of life that children learn how to eat properly, take in sufficient and balanced nutrients, regulate consumption of different food groups and develop an affinity for PA and sport exercise [21]. All of these learning experiences provide a framework for their dietary and fitness habits later in life with tremendous implications for their ability to achieve health and well-being. Children who don’t acquire sound nutritional practices and who don’t practice PA routinely put themselves at risk for a wide range of chronic diseases, many of which can increase their risk of premature mortality [18,20]. Perhaps the greatest influence on a child’s well-being and their pursuit of sound dietary practices and a healthy lifestyle is their immediate family. Parents in particular, are an important influence given their control of food purchasing, mealtime preparation of foods, and because they can role model good eating practices and exercise habits [28,30,32]. Parents are thus the bedrock on which most of a child’s pursuit of healthy eating and living starts.

There are several limitations to the current study that are worth noting. First, the sample was relatively small, drawn from a particular region in Italy, and may not be representative of the larger population of Italian school children. To test this assumption, we compared our sample with a relatively large epidemiological surveillance database for primary school children obtained from the Italian Ministry of Health (OKkio alla SALUTE, 2019). This comparison revealed that the 30% prevalence of overweight or obese conditions in our sample is consistent with the official prevalence of overweight and obesity in the Emilia-Romagna primary school population (26.4%) [26]. In addition, the KIDMED index for our sample was similar to that of other Italian primary school children’s dietary assessments [26]. With regard to PA levels, less than half (39%) of our sample met the WHO recommended criteria of at least 60 min of MVPA per day [58]. However, the majority of children in our sample (89%) reported participating in sports activities outside of school hours. Some of the noted differences in PA levels between our findings and others (including the OKkio alla SALUTE, 2019) may be attributed to measurement as we used actigraph accelerometers to accurately measure PA levels whereas other studies have relied solely on self-reports. Of note, even if the curent study took into account any form of MVPA using Actigraphs, future studies should differentiate between various physical activities (e.g., walking, cycling or gardening). When we examined self-reported sedentary behavior and, more specifically, leisure screen time, slightly under one-half of our sample exceeded 100 daily minutes.

Dietary intake is based on portion size and we are aware that overestimation of portion size can result in an overestimation of calorie and nutrient intake, leading to potential miscalculation in the assessment of diet. Self-reported dietary intake represents a limitation in our study; however for this reason we used a well-validated questionnaire with standardized portion sizes for all food items to ensure consistency in the measurement of dietary intake.

The cross-sectional nature of the data also limits us from making causal inferences regarding parental characteristics as determinants of children’s lifestyle factors. Causality requires establishing temporal relationships where the predictor precedes the outcomes in time and rigorous methodological designs with appropriate statistical controls that permit causal inferences. Notwithstanding, naturalistic observational studies such as the current one provides an important starting point to learn more about associations between variables, and this effort can be followed by prospective longitudinal studies that examine these relations over time and with appropriate statistical controls.

Finally, there are many more measures that could be modeled and that reflect parental socialization that may bear on children’s lifestyle habits. Parent-child dynamics, size of household, parent-child communication, marital status and other historical factors can influence the way parents and children interact. The omission of relevant variables can contribute to mis-specified models or at the very least produce biased parameters.

The results of this study represent a significant contribution to the field of health promotion, particularly with regards to understanding the impact of parental factors on children’s lifestyles. The novelty of our study lies in the fact that few articles have specifically investigated primary school children in Italy, and we have highlighted the unique impact of fathers’ and mothers’ factors on children’s lifestyle and nutritional habits. The results of this study have important implications for public health interventions and can help in targeting the right actors to improve children’s well-being. The insights gained from our study can be used to develop effective strategies to encourage healthy habits and promote a healthy lifestyle in children. These results have the potential to positively impact the daily lives of children and contribute to a more sustainable future for the current and next generations.

## 5. Conclusions

The current study confirms that parental factors, including lifestyle habits, educational level, and nutritional knowledge are related significantly to lifestyle factors in primary school children. This includes their dietary habits, physical activity level, and sedentary behaviors. Clearly, given these relations, it is vital that parents are included as targets in interventions, given that their adoption of healthy lifestyles will trickle down to their children. Importantly, the conduit for this “trickle down” effect can be partly attributed to the parents’ nutritional knowledge as well as their educational background and nutritional awareness. Overall, our study highlights the importance of considering the role of parental factors in shaping children’s lifestyles and suggests that targeted interventions that involve both parents and children may be an effective means of promoting healthy behaviors and reducing social inequalities in this population. Future longitudinal studies should investigate and compare different geographical areas as potential determinants of lifestyles as well as the role of teachers in influencing children’s dietary practices and healthy lifestyles. In light of this, there is a need to continue implementing school-based interventions targeting students, parents, and teachers.

## Figures and Tables

**Table 1:** Sample characteristics.

Sample Characteristic	*n* = 106
**Gender**	
Females	53 (50%)
Males	53 (50%)
**Age (mean ± SD)**	7.92 ± 1.40
**IOTF category**	
Normal Weight/Underweight	72 (68%)
Overweight/Obese	33 (32%)
Missing	1
**KIDMED Index**	4.44 ± 2.28
**KIDMED categories**	
Low	35 (33%)
Average	59 (56%)
Optimal	12 (11%)
**Average daily organized sport, minutes (mean ± SD)**	22.46 ± 16.91
**Average daily moderate-vigorous PA, minutes (mean ± SD)**	48.25 ± 17.74
**Average daily leisure screen time, minutes (mean ± SD)**	102.31 ± 33.84
**Mother educational level**	
1—High school or lower	63 (60%)
2—University degree or higher	42 (40%)
Missing	1
**Father educational level**	
1—High school or lower	78 (74%)
2—University degree or higher	27 (26%)
Missing	1
**At least one parents exercise regularly**	
No	57 (54%)
Yes	49 (46%)
**Parental Nutritional Knowledge (mean ± SD)**	5.58 ± 1.19

Note: (*n*) represents the absolute frequency and the symbol (%) represents the relative frequency. IOTF: International Obesity Task Force.

**Table 2:** Results of the Linear Regression Models.

	KIDMED			Leisure Screen Time (Average Daily Minutes)			Organized Sport (Average Daily Minutes)			MVPA (Average Daily Minutes of Physical Activity)		
	Beta	95%CI	*p*-Value	OR	95%CI	*p*-Value	Beta	95%CI	*p*-Value	Beta	95%CI	*p*-Value
**Age (years)**	0.17	−0.13, 0.46	0.269	1.26	1.02, 1.73	**0.04**	3.5	1.3, 5.7	**0.002**	−25	−42, −8.1	**0.004**
**Gender**												
*Females*	—	—		—	—		—	—		—	—	
*Males*	0.54	−0.28, 1.4	0.194	0.86	0.36, 2.07	0.7	8.1	2.0, 14	**0.010**	82	36, 129	**0.001**
**Mother educational level**												
*High School or lower*	—	—		—	—		—	—		—	—	
*University degree or higher*	0.43	−0.58, 1.4	0.404	0.29	0.10, 0.81	**0.019**	−4.9	−12, 1.9	0.153	−3.3	−55, 49	0.900
**Father educational level**												
*High School or lower*	—	—		—	—		—	—		—	—	
*University degree or higher*	1.0	0.10, 1.9	**0.030**	0.86	0.34, 2.21	0.7	1.1	−6.3, 8.5	0.769	23	−34, 80	0.423
**Parental Nutritional knowledge**	0.45	0.10, 0.81	**0.013**	0.57	0.22, 1.41	0.2	2.8	0.15, 5.4	**0.039**	14	−5.9, 34	0.166
**Sport participation (parental)**												
*No*	—	—		—	—		—	—		—	—	
*Yes*	1.0	0.17, 1.9	**0.020**	0.8	0.32, 2.00	0.6	0.98	−5.6, 7.5	0.767	7.2	−42, 57	0.773

Notes. Beta = unstandardized regression coefficient. Parameters adjusted for the presence of any other variables in the models. Bold *p*-values indicate statistical significance at the *p* < 0.05 level.

**Table 3:** Components of Diet Quality Index-International (DQI-I), percentage of the study population in component subcategories and respective scores.

Component and Points	Full Score	Scoring Criteria	N (%)	Mean ± SD
**DQI-M, total**	0–100			53.89 ± 8.69
**Variety**	0–20			10.21 ± 3.41
*Overall food group variety*	0–15			9.03 ± 2.78
0		None from any food group	0 (0%)	
3		≥4 food groups missing/d	4 (3.6%)	
6		Any 3 food group missing/d	30 (27%)	
9		Any 2 food group missing/d	44 (39%)	
12		Any 1 food group missing/d	29 (26%)	
15		≥1 serving from each food group/d	5 (4.5%)	
*Within-group variety for protein sources*	0–5			1.18 ± 1.01
0		None	20 (18%)	
1		From 1 source/d	74 (66%)	
3		2 different sources/d	16 (14%)	
5		≥3 different sources/d	2 (1.8%)	
**Adequacy**	0–40			27.13 ± 5.64
*Grain group*	0–5			3.80 ± 1.58
0		0% recommendations	0 (0%)	
1		<50% recommendations	21 (19%)	
3		50–100% recommendations	25 (22%)	
5		>100% recommendations	66 (59%)	
*Vegetable group*	0–5			1.25 ± 0.99
0		0% recommendations	12 (11%)	
1		<50% recommendations	83 (74%)	
3		50–100% recommendations	14 (12%)	
5		>100% recommendations	3 (2.7%)	
*Fruit group*	0–5			2.24 ± 1.87
0		0% recommendations	1 (0.9%)	
1		<50% recommendations	76 (68%)	
3		50–100% recommendations	0 (0%)	
5		>100% recommendations	35 (31%)	
*Fiber*	0–5			4.21 ± 1.32
0		0% recommendations	0 (0%)	
1		<50% recommendations	11 (9.8%)	
3		50–100% recommendations	22 (20%)	
5		>100% recommendations	79 (71%)	
*Protein*	0–5			4.88 ± 0.49
0		0% recommendations	0 (0%)	
1		<50% recommendations	0 (0%)	
3		50–100% recommendations	7 (6.2%)	
5		>100% recommendations	105 (94%)	
*Iron*	0–5			3.52 ± 1.13
0		0% recommendations	0 (0%)	
1		<50% recommendations	7 (6.2%)	
3		50–100% recommendations	69 (62%)	
5		>100% recommendations	36 (32%)	
*Calcium*	0–5			2.68 ± 1.41
0		0% recommendations	0 (0%)	
1		<50% recommendations	38 (34%)	
3		50–100% recommendations	54 (48%)	
5		>100% recommendations	20 (18%)	
*Vitamin C*	0–5			4.55 ± 1.03
0		0% recommendations	0 (0%)	
1		<50% recommendations	5 (4.5%)	
3		50–100% recommendations	15 (13%)	
5		>100% recommendations	92 (82%)	
**Moderation**	0–30			13.02 ± 3.52
*Total fat*	0–6			0.51 ± 1.50
0		>35% of total energy/d	99 (88%)	
3		30–35% of total energy/d	7 (6.2%)	
6		≤30% of total energy/d	6 (5.4%)	
*Saturated fat*	0–6			0.96 ± 1.41
0		>10% of total energy/d	76 (68%)	
3		7–10% of total energy/d	36 (32%)	
6		≤7% of total energy/d	0 (0%)	
*Cholesterol*	0–6			5.22 ± 1.60
0		>400 mg/d	5 (4.5%)	
3		300–400 mg/d	19 (17%)	
6		≤300 mg/d	88 (79%)	
*Sodium*	0–6			5.68 ± 1.16
0		>3400 mg/d	3 (2.7%)	
3		2400–3400 mg/d	6 (5.4%)	
6		≤2400 mg/d	103 (92%)	
*“Empty calorie foods”*	0–6			0.64 ± 1.42
0		>10% of total energy/d	91 (81%)	
3		3–10% of total energy/d	18 (16%)	
6		≤3% of total energy/d	3 (2.7%)	
**Overall balance**	0–10			3.54 ± 1.34
*Macronutrients ratio (carbohydrate:protein:fat)*	0–6			3.29 ± 1.31
0		Otherwise	11 (9.8%)	
2		50–70:8–17:12–35	18 (16%)	
4		65–68:9–16:13–32	83 (74%)	
6		55–65:10–15:15–30	0 (0%)	
*Fatty acids ratio (PUFA + MUFA)/SFA*	0–4			0.25 ± 0.93
0		<1.7	102 (91%)	
2		1.7–2	8 (7.1%)	
4		>2	2 (1.8%)	

DQI-I: Diet Quality Index-International.

## Data Availability

The data presented in this study are available on request from the corresponding author. The data are not publicly available due to ethical and privacy reasons.

## Supplementary Materials

The following supporting information can be downloaded at: https://www.mdpi.com/article/10.3390/ijerph20054240/s1. Table S1. Results of Analysis of Variance Predicting KIDMED index, Leisure Screen Time, Organized Sports, and Physical Activity. Table S2. Results of Univariate Analyses with Continuous Variables Predicting KIDMED Dietary Adherence Score. Table S3. Results of Univariate Analyses with Continuous Variables Predicting Leisure Screen Time (daily minutes). Table S4. Results of Univariate Analyses with Continuous Variables Predicting Minutes of Organized Physical Activity. Table S5. Results of Univariate Analyses with Continuous Variables Predicting Minutes of Vigorous Physical Activity.
