# Supplementary material for: Cross-Sectional Analysis of Family Factors Associated with Lifestyle Habits in a Sample of Italian Primary School Children: The I-MOVE Project
Source: Int J Environ Res Public Health. 2023 Feb 27;20(5):4240. doi: 10.3390/ijerph20054240 (PMC10002146; doi:10.3390/ijerph20054240)
Supplement: Supplementary file 1 [file ijerph-20-04240-s001.zip › ijerph-2218373-supplementary.pdf]

Supplemental Materials

Table S1. Results of Analysis of Variance Predicting KIDMED index, Leisure Screen Time, Organized Sports and Physical Activity.

	KIDMED		Leisure Screen Time (Average Daily Minutes)		Organized Sports (Average Daily Minutes)		Physical Activity		
**Measure**	**Mean±SD**	***p*-Value**	**Mean±SD**	***p*-Value**	**Mean±SD**	***p*-Value**	**Mean±SD**	***p*-Value**	**Sample Size**
Gender		0.300		0.8		**0.008**		**0.002**	53/53
Boys	4.72±2.45		95.96±28.69		26.78±15.66		376.01±129.66		
Girls	4.21±2.29		94.64±38.66		18.14±17.14		301.03±107.55		
Education - Mother		**0.024**						0.5	63/42
High school or lower	4.18±2.37		99.82±30.97	**0.017**	23.22±17.57	0.6	331.14±129.45		
University+	5.37±2.20		81.59±39.34		21.24±16.21		347.60±118.52		
Education - Father		**<0.001**		0.3		0.948		0.9	78/27
High school or lower	3.79±2.09		97.96±35.03		22.46±18.05		336.83±125.99		
University+	5.52±2.42		91.11±32.33		22.33±13.75		340.68±123.64		
Sport parents		**<0.001**		0.4		0.6		0.9	57/49
No Involvement	3.72±2.29		97.74±30.34		21.65±17.11		338.81±131.48		
Yes Involved	5.33±2.19		92.41±37.69		23.40±16.80		336.54±116.06		

Notes: Variables are entered one by one. Bold *p*-values indicate statistical significance at the $p < 0.05$ level.

Table S2. Results of Univariate Analyses with Continuous Variables Predicting KIDMED Dietary Adherence Score.

**Characteristic**	**βeta**	**95% CI**	***p*-Value**
Age	0.23	−0.10, 0.56	0.2
Nutritional knowledge	0.60	0.23, 0.97	**0.002**

Note: βeta = unstandardized regression coefficient; CI = Confidence Interval. Bold *p*-values indicate statistical significance at the $p < 0.05$ level.

Table S3. Results of Univariate Analyses with Continuous Variables Predicting Leisure Screen Time (daily minutes).

**Characteristic**	**βeta**	**95% CI**	***p*-Value**
Age	4.9	0.26, 9.5	**0.039**
Nutritional knowledge	−2.7	−8.1, 2.8	0.3

Note: βeta = unstandardized regression coefficient; CI = Confidence Interval. Bold *p*-values indicate statistical significance at the $p < 0.05$ level.

Table S4. Results of Univariate Analyses with Continuous Variables Predicting Minutes of Organized Physical Activity.

**Characteristic**	**βeta**	**95% CI**	***p*-Value**
Age	3.6	1.4, 5.8	**0.002**
Nutritional knowledge	2.3	−0.38, 5.0	0.092

Note: βeta =un standardized regression coefficient; CI = Confidence Interval. Bold *p*-values indicate statistical significance at the $p < 0.05$ level.

Table S5. Results of Univariate Analyses with Continuous Variables Predicting Minutes of Vigorous Physical Acitivty.

**Characteristic**	**βeta**	**95% CI**	***p*-Value**
Age	−22	−39, −4.3	**0.015**
Nutritional knowledge	14	−6.0, 35	0.2

Note: βeta =unstandardized regression coefficient; CI = Confidence Interval; Bold *p*-values indicate statistical significance at the $p < 0.05$ level.
